# Left anterior descending artery aneurysm in a young patient with familial retinal arterial macroaneurysm: A case report

**DOI:** 10.1016/j.ajoc.2022.101548

**Published:** 2022-04-29

**Authors:** Husaam Haidar, Ahmed F. Alohali, Abdulaziz S. Albaradai, Mohammed Alreshidan, Mohmmed A. Algamdi

**Affiliations:** a Intensive Care Department, Aseer Central Hospital, Abha, Saudi Arabia; b Critical Care Services Administration, King Fahad Medical City, Riyadh, Saudi Arabia; c Adult Cardiovascular Surgery, King Fahad Medical City, Riyadh, Saudi Arabia; d Pulmonary Section, Department of Medicine, King Fahad Medical City, Riyadh, Saudi Arabia

## Abstract

**Purpose:**

Familial retinal arterial macroaneurysm (FRAM) is a rare genetic disorder caused by a gene mutation in the insulin-like growth factor binding protein 7 (IGFBP7).

**Observations:**

We report a 30-year-old male with FRAM and IGFBP7 gene mutation who presented with an acute coronary syndrome (ACS). Invasive coronary angiography revealed a large aneurysm at the proximal part of the left anterior descending (LAD) artery.

**Conclusions and Importance:**

Few cases with systemic vascular involvement in patients with FRAM have been described before; however, our case represents the first documentation of a LAD artery aneurysm in a patient with FRAM and IGFBP7 gene mutation.

## 1 Introduction

Familial retinal arterial macroaneurysm (FRAM) is an autosomal recessive disorder caused by a mutation in the insulin-like growth factor binding protein 7 (IGFBP7) with upregulation of the BRAF/MEK/ERK pathway,^1^ which consists of a cascade of events that ultimately activate transcription factors resulting in gene expression. FRAM's fundoscopic features usually include unilateral or bilateral multiple retinal arterial macroaneurysms formation and arterial beading along the major retinal arterial trunks with recurrent episodes of hemorrhage and exudation.^1^ Systemic vascular involvement in patients with FRAM is exceedingly rare.^1^^,^^2^ This report presents the first case of a LAD artery aneurysm in a patient with familial retinal arterial macroaneurysm.

## 2 Case presentation

A 30-year-old man presented to our hospital with seven days history of exertional chest pain. The pain was increasing in its intensity but not associated with cough, dyspnea, or fever. Asides from active cigarette smoking, the patient denied substance abuse and exposure to sick contacts or new medications. The patient denied a history of hypertension, diabetes, or connective tissue disease. His past medical history was only remarkable for left eye blindness due to a left retinal artery macroaneurysm related to his IGFBP7 gene mutation for seven years ago. The patient's three brothers and one sister were previously screened for the IGFBP7 gene mutation, and only one of the brothers carries the gene abnormality and has eye manifestations.

**Fig. 1:**
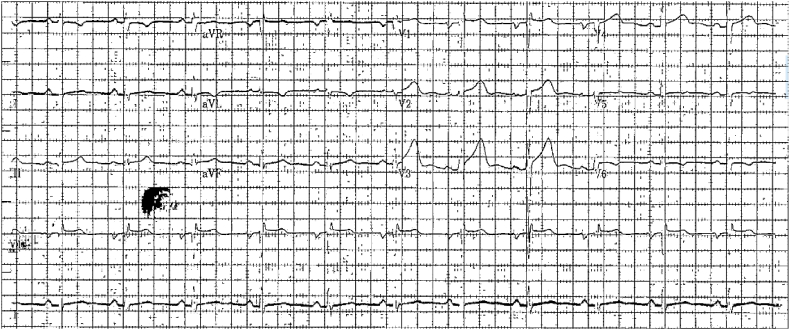
An electrocardiogram demonstrating non-specific ST-T wave changes.

Initial investigation included a chest X-ray (CXR), which demonstrated no abnormalities, an electrocardiogram, which demonstrated non-specific changes (Fig. 1), and an elevated troponin level. The patient was admitted through the Emergency Department as a case of acute coronary syndrome (ACS) with stable hemodynamic parameters and was started on dual antiplatelet drugs and venous thromboembolism prophylaxis.

**Fig. 2:**
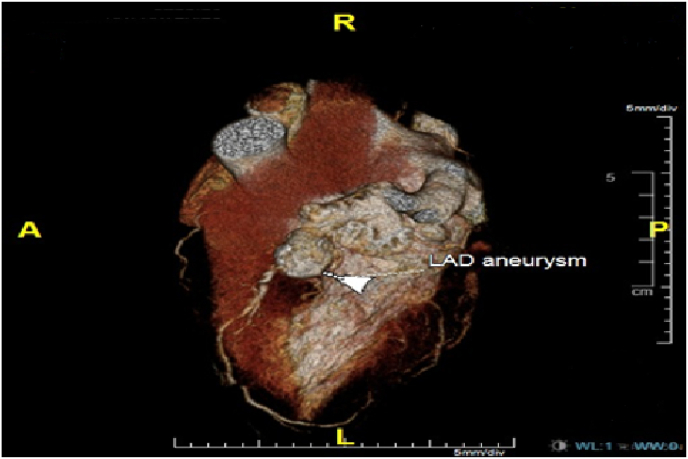
A three-dimensional construction of cardiac computerized tomography scan showing the LAD aneurysm.

**Fig. 3:**
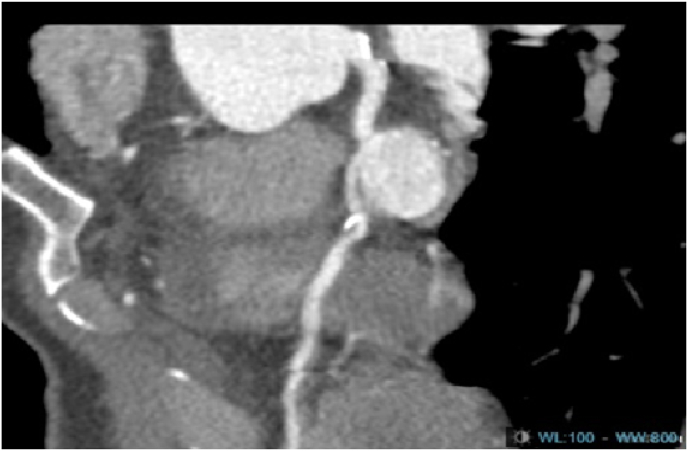
A computerized tomography coronary angiogram showing the LAD aneurysm.

Initial diagnostic intervention consisted of coronary computed tomography (CT) angiography, which revealed a large aneurysm (1.5 × 1.8 cm) at the proximal part of left anterior descending (LAD) artery associated with peripheral calcification (Fig. 2, Fig. 3). Additionally, invasive coronary angiography showed a large LAD artery aneurysm and a small non-dominant left circumflex artery associated with miniature second obtuse marginal with a severe stenotic lesion distally. Invasive coronary angiography of the right coronary artery showed mild proximal to mid segment disease with minor calcifications. CT angiography of the brain, chest, abdomen, and pelvis did not reveal any aneurysmal dilatation in cerebral, aortic or major arterial branches.

After a multidisciplinary discussion including interventional cardiology and cardiac surgery teams, a decision was made to proceed with an open-heart surgery. Finding during the operation included an atheromatous and thickened wall of the LAD artery with associated giant aneurysm, measuring approximately 2 cm in diameter, with scattered calcifications. Ligation of the LAD artery, proximal and distal to the aneurysm was done, with obliteration of the aneurysm with pledged sutures. The LAD was bypassed using a great saphenous vein since the LAD artery wall was of small lumen and extremely thickened. The saphenous vein graft was used to serve as a high flow conduit to prevent any ischemia to the anterior wall of the heart.

At the conclusion of the procedure, the patient could not be weaned from the cardiopulmonary bypass machine. The intraoperative *trans*-esophageal echocardiography (TEE) revealed distended right and left ventricles with global hypokinesia of both ventricles. We tried to de-air the graft, but the heart continued to appear stunned; hence, an intra-aortic balloon pump (IABP) was inserted followed by a central Veno-Arterial Extracorporeal Membrane Oxygenation (VA-ECMO). In the immediate postoperative period, the patient continued to demonstrate a hemodynamic instability in the Cardiovascular Intensive Care Unit (CVICU), thereby, the patient underwent an emergency coronary angiography. A Percutaneous Coronary Intervention to the right coronary artery was done due to proximal dissection and a minimal flow into the aneurysm was recognized, which was sealed off to eliminate the risk of proximal propagation of the thrombosis. The patient was brought back to the CVICU in a relatively stable condition.

**Fig. 4:**
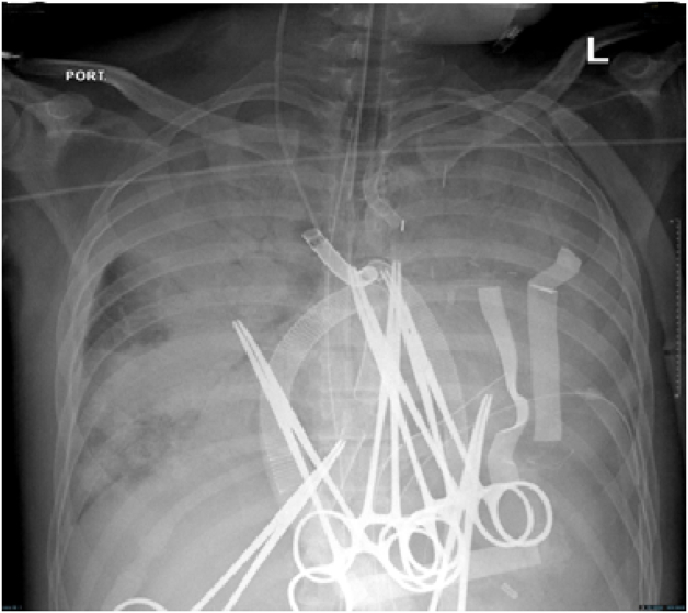
Portable CXR on the 7th day of the cardiovascular ICU admission showing bilateral and diffuse lung infiltrate. The patient was on central VA-ECMO, IAPB, and his chest was kept open with approximated skin.

**Fig. 5:**
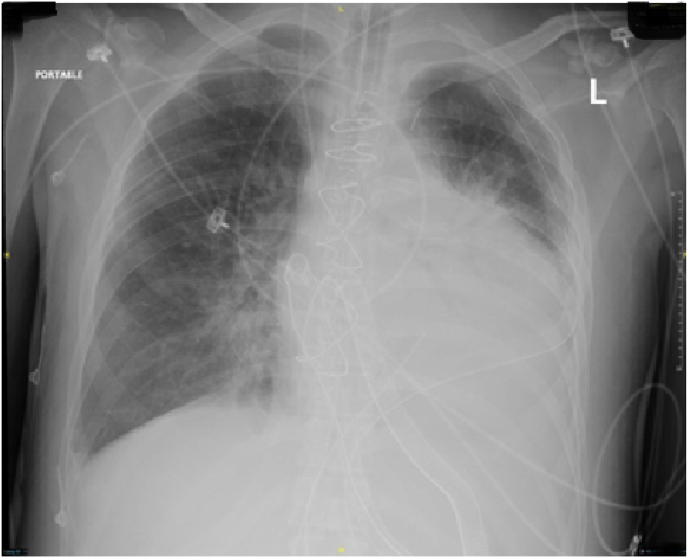
Portable CXR after three weeks of the cardiovascular ICU admission showing improvement of the bilateral lung infiltrate. The IABP was removed, the Central ECMO was converted to a CentriMag BiVAD, and the sternum was closed.

Initial management in the CVICU included deep sedation and muscle paralysis. The perfusion was maintained targeting normal urine output and lactate level. *Trans*-esophageal Echocardiography showed severely depressed left and right ventricular systolic functions. The patient developed bilateral lung infiltrate suggestive of acute respiratory distress syndrome (ARDS), which led to increased oxygen requirement and decreased lung compliance (Fig. 4). COVID-19 screening was negative two times, and bacterial cultures were also negative. The patient was empirically covered with antibiotics, and protective lung ventilation and restrictive fluid therapy were pursued, resulting in physiological, clinical, and radiological improvement within two weeks of therapy (Fig. 5). The IABP was removed and the Central ECMO was converted to a CentriMag biventricular assist device (BiVAD). Five days later, the patient was disconnected from mechanical ventilation and the endotracheal tube was removed. Shortly, the patient was transferred to a regional transplant center for heart transplantation as a destination therapy. The Patient was successfully transplanted with a satisfactory post-transplant recovery and was subsequently discharged home.

## 3 Discussion

LAD artery aneurysm is a rare condition affecting 0.3–4.9% of patients undergoing coronary angiography. Common causes of LAD artery aneurism include vasculitis, atherosclerosis, cocaine use, post instrumentation, and infection such as Lyme and syphilis. Coronary artery aneurisms are usually discovered incidentally upon investigating patients presenting with Acute coronary syndrome, rupture of the aneurysm, or cardiac tamponade. The right coronary artery is affected the most (40%) followed by the LAD artery (32%) and the left main artery (3.5%). The usual work up includes echocardiography, coronary (CT) angiography, and invasive coronary angiography. Treatment varies and ranges from medical therapy with antiplatelet drugs, percutaneous coronary intervention, and open-heart surgery.^3^^,^^4^

In 2002, Dhindsa and Abboud, described the clinical features and the course of a new clinical entity which they called, familial retinal arterial macroaneurysm (FRAM). The two authors described seven patients from three different families with beading and macroaneurysms along the major retinal arterial trunks bilaterally. Of those patients, five had recurrent bleeding and macroaneurysms leakage resulting in visual loss.^5^ In 2012, Abu-Safieh and her colleagues evaluated 22 patients from 8 different Saudi families with typical features of FRAM.^1^ The authors demonstrated an autosomal-recessive mode of inheritance among examined families and causative association with a gene mutation in the insulin-like growth factor binding protein 7 (IGFBP7). Of note, supravalvular pulmonic stenosis was found in all patients who underwent echocardiography (9 out of 22 patients). Coronary artery involvement in patients with FRAM was first described by Nowilaty and her colleagues in 2014. In their case report, polyangiograms revealed several vascular abnormalities which were initially thought to be related to Takayasu disease including stenosis of the LAD coronary artery with unusual ostial coronary aneurysms, occlusion of the left subclavian artery, stenosis of both renal arteries, irregularities in the mesenteric artery and tapering of the aorta.^2^

In our report, we describe the first case of LAD artery aneurysm in a patient with familial retinal arterial macroaneurysm (FRAM) is result of the IGFBP7 gene mutation. The absence of inciting drugs, chest trauma, connective tissue disease, or infection and the patient's age increases the probability of the causative effect of his IGFBP7 gene mutation. Furthermore, the patient's brother, who tested positive for the IGFBP7 gene mutation, was found to have a right coronary artery aneurism upon family screening. Our case demonstrates the possibility of developing LAD artery aneurysm in patients with familial retinal arterial macroaneurysm (FRAM) and provides further evidence of the systemic vascular involvement in such patients. The LAD aneurysm in our patients resulted in myocardial infarction which was complicated with post-operative severe cardiogenic shock, ultimately, requiring heart transplantation as a distant therapy. The potential devastating outcomes in some patients with FRAM signify the importance of a thorough and careful systematic vascular review. In addition, cardiac surgeons should be cautious while operating on these patients and be prepared for possible difficulties as the aneurysm may also be accompanied by other vessel wall abnormalities.

## Patient consent

Written consent to publish this case has not been obtained. This report does not contain any personal identifying information.

## Funding

No funding or grant support.

## Authorship

All authors attest that they meet the current ICMJE criteria for Authorship.

## Declaration of competing interest

All the authors have no financial disclosures.
